# Comment on Klek et al. Enhanced Recovery after Surgery (ERAS) Protocol Is a Safe and Effective Approach in Patients with Gastrointestinal Fistulas Undergoing Reconstruction: Results from a Prospective Study. *Nutrients* 2021, *13*, 1953

**DOI:** 10.3390/nu14010017

**Published:** 2021-12-22

**Authors:** Augusto Lauro, Maria Cristina Ripoli

**Affiliations:** 1 Department of Surgical Sciences “F. Durante”, Sapienza University, 00185 Rome, Italy; 2 Ospedale “Ceccarini” di Riccione, 47838 Rimini, Italy; cristina-ri@hotmail.it

We read and appreciated the prospective study by Klek et al. [1] recently published in *Nutrients*: an ERAS (Enhanced Recovery After Surgery) protocol was used by the authors to reduce post-operative complications in all subjects scheduled for surgery due to entero-cutaneous fistulas, collecting a consecutive series of 100 patients between 2011 and 2020. The ERAS protocol was successfully applied even though few modifications of the original plan were introduced in 2015, dividing the patients in two groups (2011–2015 and 2016–2020): a statistically significant improvement of surgical outcomes was shown by the authors, reporting a reduction of postoperative nausea and vomiting, overall complication rate, and median length of hospital stay (overall and after surgery).

We do agree that ERAS protocols have demonstrated their efficacy after colorectal surgery [2], but the unquestionable novelty of the study by Klek et al. requires further discussion. Surgery for entero-cutaneous fistulas represents a challenge [3,4]: in our previous systematic review of the literature on this topic [5], we included in our study 1217 patients. A bowel resection with primary anastomosis was performed in 1048 patients, and 856 patients (81.7%) had a fistula takedown in one procedure without a temporary covering stoma. The patients had 14.3% recurrence and 13.1% mortality rate respectively.

Our question to the authors is related to their surgical technique. They [1] reported a 0% mortality rate in both groups and an overall complication rate of 34.4% and 14.5% in group 1 (2011–2015) and group 2 (2016–2020), respectively. Their outcome is clearly improved compared to international literature and to their initial series along the years, but it would be very interesting to let the reader know if they performed a temporary stoma to cover their anastomoses or not.

The matter does not represent a futile topic under the clinical point of view: a staged operation [6] or primary anastomosis without stoma [5] could determine relevant implications on the patient’ recovery, and these factors should be considered to fully evaluate the modified ERAS protocol as safe and effective in patients treated for gastrointestinal fistulas.

We thank in advance the authors for their kind reply and congratulate them for their pioneering report in such a demanding and controversial field.
